# Magnesium valproate adjuvant therapy on patients with dementia: A protocol for systematic review and meta-analysis

**DOI:** 10.1097/MD.0000000000028161

**Published:** 2021-12-23

**Authors:** Chen qi Zhang, ke xin Zheng, Ling qi Sun, Hongbin Sun

**Affiliations:** a Department of Special Medical, Chengdu BOE hospital, Chengdu, Sichuan Province, China; b Department of Neurology, The Air Force Hospital of Western Theater Command, Chengdu, Sichuan Province, China; c Department of Neurology, Sichuan academy of medical sciences and Sichuan. provincial people's hospital, Chengdu, China.

**Keywords:** dementia, magnesium valproate, meta-analysis

## Abstract

**Background:**

With the aging population, the prevalence and incidence of dementia disease will continue to rise, and the associated economic burden is increasing as well. However, the available anti-dementia therapeutic arsenal is limited. Meanwhile, magnesium valproate (VPM) as an adjuvant therapy had a general positive effect on the cognitive function and psychiatric symptoms of patient with dementia (PwD). At present, there is lack of meta-analysis focusing on cognitive improvement and disease-modifying about VPM-assisted therapy in the present peer-reviewed literature. Thus, we aimed to likely analyze the efficacy and safety of VPM adjuvant therapy of PwD.

**Methods:**

We will research MEDLINE via PubMed, Cochrane Library, EBSCO, Embase, China National Knowledge (CNKI) and Wan fang databases to gather relevant data on VPM assistant therapy on the PwD. Meta-analysis will be performed using Stata16.0 software.

**Results:**

We aim to evaluate the efficacy and safety of VPM in the adjuvant treatment of PwD.

**Conclusion:**

VPM maybe plays an active role in the treatment of dementia patients and this research will provide reliable evidence for clinicians in therapy of PwD.

**INPLASY registration number:**

INPLASY2021110038 (DOI: 10.37766/inplasy2021.11.0038).

## Abstract 2

Supplemental Digital Content is available in the text

## 1 Introduction

According to US population estimate of people with clinical Alzheimer disease (AD) and mild cognitive impairment, an estimated 6.2 million Americans aged 65 and older are living with AD today. By 2060, number could grow to 13.8 million.^[1]^ Dementia affects individuals, their families, and the social economy, its costs estimated at about US$1 trillion annually.^[2]^ In addition, the cost also includes an increased risk of emotional distress and negative physical and mental health outcomes for family caregivers.^[3]^ These figures reflect dementia patients have a higher burden of illness compared with other disease.

However, there is no pharmacological treatment presently for dementia that can delay or stop the damage and destruction of neurons, which is the reason of Alzheimer's symptoms and make the disease fatal.^[3]^ According to the in vitro and in vivo studies, valproate (VPA), a commonly prescribed antiepileptic drug,^[4]^ may have neuroprotective effects on PwD, through a variety of potential mechanisms including actions on gamma-aminobutyric acid and N-methyl-d-aspartate receptors, prevention of beta- amyloid aggregation, decreased beta amyloid and neurotic plaque production, and induction of neurogenesis to ameliorate the symptoms of dementia.^[5]^

VPM is a salt form of valproate which combined with magnesium.^[6]^ In clinical and laboratory studies, a decreased magnesium concentration was found in various tissues of PwD, including cerebral spinal fluid, red blood cells, plasma, and hair,^[7]^ reduced magnesium levels in the hippocampus particularly, seem to be an important factor in the pathogenesis of AD. There is new support for the neuroprotective effect of magnesium based on animal studies, suggesting that magnesium treatment at the early stage for dementia patients may delay their cognitive decline.^[8,9]^ However, magnesium for its ability to affect vascular function in addition to neuronal function.^[10]^ Thus, based on these theories, VPM may be affecting cognitive function in multiple distinct ways.

At present, many controlled studies using VPM as complementary therapy showed positive effects with dementia patients.^[11–14]^ Although there are some experiments showed negative results of VPA for dementia patients, most of these tests used VPA as a mono therapy to compare with the placebo group. Hence, present research has found contradictory results on the treatment of VPM or VPA and lack of meta-analysis focusing on VPM-assisted therapy in the PwD in the present peer-reviewed literature. It requires further investigation and standardized ways to evaluate the effects of VPM in dementia patients. Thus, we aim to likely analyze the efficacy and safety of VPM adjuvant therapy of PwD based on RCTs.

## 2 Methods

### 2.1 Study registration

This present study was registered on the International Platform of Registered Systematic Review and Meta-analysis Protocols with the registration number of INPLASY2021110038 and the DOI number is 10.37766/inplasy2021.11.0038 (https://inplasy.com/inplasy-2021–11–0038/). This research will be conducted in accordance with the guidelines of the Preferred Reporting Items for Systematic Reviews and Meta-Analyses Protocols statement guidelines.^[15]^

### 2.2 Selection criteria

#### 2.2.1 Participants

We will include participants diagnosed with dementia by any proper clinical criteria. There is no restriction on age, sex, race, treatment time, and region of the enrolled patients.

#### 2.2.2 Interventions and comparators

The control group was defined as patients who were treated to conventional anti-dementia medicine and the treatment group will be assigned with oral VPM-plus conventional anti-dementia medicine which must be the same as the control group.

#### 2.2.3 Outcomes

The primary outcome indicators will be clinical efficacy (the change in cognitive function scores derived from MMSE, ADAS-cog and MOCA scales). The secondary outcome indicators will be efficacy in psychiatric effects include assessments of psychological symptoms of dementia which might be measured by the BRMS, Neuropsychiatric Inventory , Cohen-Mansfield Agitation Inventory, and other scales. The Incidences of adverse events associated to VPM will also be included as secondary outcomes.

#### 2.2.4 Study design

We include randomized controlled trials which provided detailed and clear outcome of interest. Article of non- randomized controlled trials, observational studies, case reports, reviews, and studies which has unclear outcome data will be exclude. Even though we do not have restrictions on language, but the search object was restricted to human.

### 2.3 Search strategy

MEDLINE via PubMed, Cochrane Library, EBSCO, Embase, China National Knowledge (CNKI) and Wan fang databases (up to October, 2021) will be applied for preliminary literature screen. We will conduct by using medical subject headings (Mesh) and term words, such as “Valproate Magnesium” [Mesh], “Valproic acid magnesium,” “Magnesium dipropyl acetate,” “Dementia” [Mesh], “dement∗,” “Alzheimer∗,” “Huntington∗,” and so on. Additionally, we will also manually check all references relevant to the included studies to avoid inappropriate omissions.

### 2.4 Study selection and data extraction

The Endnote X9 literature management software will be applied for all procession of the screening records. Two researchers will separately check the topics, titles and abstract according to inclusion and exclusion criteria. If there exists any discrepancies between the 2 authors, we resolved it by discussion or consulting with the senior reviewer. The extracted data will include the first author's name, publication date, sample sizes, mean age, sex, details of participants, diagnostic criteria, treatment and control intervention, duration time, main outcome measures, and adverse events.

### 2.5 Risk of bias assessment

Two reviewers respectively used bias risk assessment guideline (recommended by the Cochrane handbook http://community.cochrane.org/handbook) for quality assessment. Differences will be discussed with a third researcher until consensus is reached. The domains including the following 7 aspects, include random sequence generation, allocation concealment, blinding of the participants and personnel, blinding of the assessors, incomplete outcome data, and selective outcome reporting.

### 2.6 Statistical analysis

We will perform statistical analyses using Stata 16.0 (Stata Corporation, College Station, TX) software. Standardized mean difference (SMD) with the 95% CI as an effect size was measured for continuous data. As far as dichotomous data, the risk ratios (RRs) with 95% CI was calculated. Cochran's Q statistic and I2 metric statistics were used to assess the level of heterogeneity. If I2 <50%, the heterogeneity is small, data will be analyzed using a fixed-effects model. If I2>50% and P < 0.05, data will be analyzed using a random effects model. If necessary, a leave-one-out sensitivity analysis and subgroup analysis will be performed to evaluate the main trials demonstrating a substantial impact on the inter-study heterogeneity.

### 2.7 Confidence in cumulative evidence

The Grading of Recommendations Assessment will be performed for evaluating the analysis results. The domains including the following five aspects (risk of bias, indirectness, inconsistency, imprecision and publication bias) which be divided into four levels: very low, low, moderate and high.

### 2.8 Ethics and dissemination

Our study did not require ethical approval because patients and human trials were not involved.

## 3 Discussion

Learned from 2020 report of the Lancet Commission, there are about 50 million people living with dementia worldwide, especially in low-income and middle- income countries, and that number is expected to rise to 152 million by 2050.^[2]^ Currently, the US Food and Drug Administration (FDA) has allowed 5 drugs for the treatment of AD until 2020: rivastigmine, galantamine, donepezil, memantine, and memantine combined with donepezil now, but none of these medicines are approved to treat behavioral and psychiatric symptoms of PwD.^[3]^ These findings emphasize the urge to consider increase more potential effective medicine in the dementia-associated clinical trials.

Although meta-analysis has proved that low-dose VPA is effective in treating agitation among demented patients, and that high-dose VPA is associated with an unacceptable rate of adverse effects.^[5,10]^ There is new support for the neuroprotective effect of magnesium based on animal studies, suggesting that magnesium treatment at the early stage for dementia patients may delay their cognitive decline.^[8,9]^

A positive effect of VPM (a salt form of VPA) has been reported in several clinical studies. However, conventional meta-analysis can only perform VPA as a complement therapy, and there is lack of meta-analysis focusing on cognitive improvement and disease-modifying about VPM-assisted therapy in the current peer-reviewed literature. As far as we know, this is the first time that VPM has been used as an adjuvant therapy to treat PwD. we aimed to likely analyze the efficacy and safety of VPM adjuvant therapy to help in the treatment of PwD and provide direct evidence supporting this conclusion (Supplementary Content, http://links.lww.com/MD/G524).

## Author contributions

**Conceptualization:** Chen-qi Zhang, Hong-bin Sun.

**Data analysis:** Chen-qi Zhang, Ke-xin Zheng, Ling-qi Sun.

**Data curation:** Chenqi Zhang, KeXin Zheng, Lingqi Sun.

**Formal analysis:** Chenqi Zhang.

**Investigation:** Chenqi Zhang, KeXin Zheng.

**Methodology:** Chenqi Zhang, Lingqi Sun, Hongbin Sun.

**Software:** Chenqi Zhang, KeXin Zheng.

**Study design:** Chen-qi Zhang, Ling-qi Sun, Hong-bin Sun.

**Supervision and Validation:** Ling-qi Sun, Hong-bin Sun.

**Supervision:** Hongbin Sun.

**Validation:** Lingqi Sun.

**Writing – original draft:** Chenqi Zhang.
